# Gene Expression and Metabolome Analysis Reveals Anti-Inflammatory Impacts of 11,17diHDoPE on PM10-Induced Mouse Lung Inflammation

**DOI:** 10.3390/ijms25105360

**Published:** 2024-05-14

**Authors:** Uijin Kim, Dong-Hyuk Kim, Deok-Kun Oh, Ha Youn Shin, Choong Hwan Lee

**Affiliations:** 1 Department of Biomedical Science & Engineering, Konkuk University, Seoul 05029, Republic of Korea; rladmlwls135@naver.com; 2 Department of Bioscience and Biotechnology, Konkuk University, Seoul 05029, Republic of Korea; walking99@konkuk.ac.kr (D.-H.K.); deokkun@konkuk.ac.kr (D.-K.O.)

**Keywords:** particulate matter, pulmonary inflammation, inflammatory cytokine genes, metabolomics, oxylipins, pro-resolving mediators, anti-inflammation, macrophage differentiation

## Abstract

Oxylipins, the metabolites of polyunsaturated fatty acids, are vital in regulating cell proliferation and inflammation. Among these oxylipins, specialized pro-resolving mediators notably contribute to inflammation resolution. Previously, we showed that the specialized pro-resolving mediators isomer 11,17dihydroxy docosapentaenoic acid (11,17diHDoPE) can be synthesized in bacterial cells and exhibits anti-inflammatory effects in mammalian cells. This study investigates the in vivo impact of 11,17diHDoPE in mice exposed to particulate matter 10 (PM10). Our results indicate that 11,17diHDoPE significantly mitigates PM10-induced lung inflammation in mice, as evidenced by reduced pro-inflammatory cytokines and pulmonary inflammation-related gene expression. Metabolomic analysis reveals that 11,17diHDoPE modulates inflammation-related metabolites such as threonine, 2-keto gluconic acid, butanoic acid, and methyl oleate in lung tissues. In addition, 11,17diHDoPE upregulates the LA-derived oxylipin pathway and downregulates arachidonic acid- and docosahexaenoic acid-derived oxylipin pathways in serum. Correlation analyses between gene expression and metabolite changes suggest that 11,17diHDoPE alleviates inflammation by interfering with macrophage differentiation. These findings underscore the in vivo role of 11,17diHDoPE in reducing pulmonary inflammation, highlighting its potential as a therapeutic agent for respiratory diseases.

## 1. Introduction

One of the environmental threats to public health stems from air pollution due to global industrialization. Particularly, air pollution is predominantly caused by the suspension of particulate matter (PM) in the air [1,2,3,4]. Due to their small size, PM with a diameter of 10 microns or smaller is referred to as PM10 and can be deeply inhaled into the lungs, potentially severely impacting human health. Although an increasing number of studies reported PM10 as a risk factor for various respiratory illnesses, the detailed underlying mechanisms of PM10-induced inflammation remain incompletely understood, and specific treatments for PM10-induced lung inflammation are yet to be established. Oxylipins, natural metabolites produced by the human body [5,6,7], emerge as a potential therapeutic candidate. Several pieces of clinical evidence have shown that levels of both pro-inflammatory and anti-inflammatory oxylipins are altered in response to inflammatory stimuli such as PM10 [8]. Although the in vivo anti-inflammatory effects of exogenous oxylipin treatment require further extensive investigation, oxylipins could serve as potential biomarkers and therapeutic agents to treat respiratory diseases caused by PM10.

Oxylipins, metabolites of polyunsaturated fatty acids oxidized by cyclooxygenase (COX), lipoxygenase (LOX), and cytochrome P450 (CYP), and play various roles, including regulation of cell proliferation, tissue repair, blood clotting, inflammation, and immunity [9]. In animals, oxylipins derived mainly from C20- and C22-polyunsaturated fatty acids are classified as lipid mediators. Among lipid mediators, C22 di- and trihydroxy fatty acids in the human body are termed specialized pro-resolving mediators (SPMs), which are involved in resolving inflammation and infection in humans [10]. Our previous study has demonstrated that one of the SPM isomers, 11,17diHDoPE, can be synthesized from docosapentaenoic acid (DPA) by arachidonate (ARA) 9S-lipoxygenase in the bacterium *Sphingopyxis macrogoltabida* [11]. We also showed that 11,17diHDoPE treatment reduced the release of lipopolysaccharide-induced cytokines, including interleukin-6 (IL-6), tumor necrosis factor-alpha (TNF-α), and IL-1 beta (IL-1β) in mammalian cells, indicating its anti-inflammatory activity. Additionally, 11,17diHDoPE treatment reduced the production of reactive oxygen species in PM10-stimulated human keratinocyte cells. This reduction was achieved by restoring the expression levels of genes responsible for scavenging reactive oxygen species, which were previously diminished due to PM10 exposure [8]. However, the in vivo role of 11,17diHDoPE in inflammatory responses remains undetermined in animal studies.

In the present study, we utilized PM10 to induce inflammation in mouse lung tissue, aiming to elucidate the in vivo effects of 11,17diHDoPE on inflammatory responses. Using integrative analysis of gene expression changes and metabolite alterations in both mouse lung tissues and sera, we demonstrated that 11,17diHDoPE can alleviate inflammatory responses by regulating the levels of inflammation-related genes and metabolite subsets. Our findings provide a fundamental understanding of how 11,17diHDoPE contributes to resolving inflammation in the lungs of mice exposed to PM10. This knowledge contributes to the development of potential therapies for respiratory diseases.

## 2. Results

### 2.1. Intranasal Pretreatment with 11,17diHDoPE Alleviates Lung Inflammation in Mice Exposed to PM10

**Figure 1:**
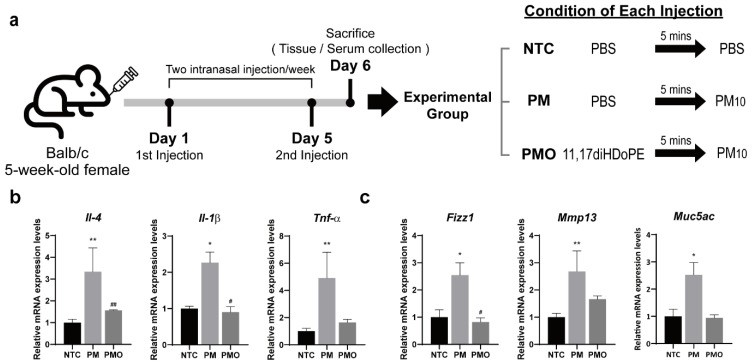
Anti-inflammatory effects of 11,17diHDoPE in mouse lung tissue exposed to PM10. (**a**) Experimental design to assess the effect of 11,17diHDoPE on inflammatory response in PM10-exposed mice. Intranasal administration of 11,17diHDoPE or PBS was conducted, followed by PM10 exposure via the same route after 5 min. Four days later, a second identical injection was administered. The following day, lung tissue and serum were collected from each mouse group for gene expression and metabolomic analyses. (**b**) Relative mRNA levels of inflammatory cytokine genes in mouse lung tissue (NTC, *n* = 7; PM, *n* = 6; PMO, *n* = 6). mRNA levels of cytokine genes were normalized to Gapdh mRNA levels. Results are shown as mean ± standard error of the mean. Mann–Whitney test was used to evaluate the statistical significance of the differences between NTC and PM (* *p* < 0.05, ** *p* < 0.01) and between PM and PMO (# *p* < 0.05, ## *p* < 0.01). (**c**) Relative mRNA levels of genes related to respiratory illness in mouse lung tissue. NTC, non-treated control; PM, PM10 injected group; PMO, PM10, and 11,17diHDoPE co-injected group.

11,17diHDoPE was synthesized from DPA by the enzyme ARA 9S-LOX of *S. macrogoltabida* as previously described (Figure S1) [11]. To determine the in vivo role of 11,17diHDoPE on pulmonary inflammation, we used PM10 to induce inflammation in mouse lung tissue and investigated whether 11,17diHDoPE could alleviate the inflammatory response. Briefly, 5-week-old Balb/c mice were divided into three groups: non-treated control (NTC), PM10-treated group (PM), and both PM10 and 11,17diHDoPE-treated group (PMO), respectively (Figure 1a). As a negative control, the NTC group was only treated with a phosphate-buffered saline (PBS) solution. The PM group was exposed to PM10 by an intranasal route, whereas the PMO group underwent intranasal pretreatment with 11,17diHDoPE before the PM10 injection. The second administration of PM10 and 11,17diHDoPE was conducted 4 days later under the same conditions. The next day, lung tissue and serum were collected from each group of mice for analysis. Quantitative reverse transcription PCR (RT-qPCR) analyses were conducted on the mouse lung tissue to measure expression levels of genes associated with pro-inflammatory cytokines. IL-4, IL-1β, and TNF-α are pro-inflammatory cytokines that play a key role in inflammatory responses [12,13,14]. Il-4, Il-1b, and Tnf-α, key pro-inflammatory cytokines exhibited a more than 2-fold increase in expression upon PM10 administration compared to the NTC group (Figure 1b). However, pretreatment with 11,17diHDoPE remarkably reduced the expression levels of these pro-inflammatory cytokine genes, reaching levels comparable to those in the NTC group. In addition, expression levels of genes related to lung function or pulmonary diseases were assessed in mouse lung tissue. Fizz1 is an important mediator in maintaining lung homeostasis, regulating inflammation, and promoting tissue repair and remodeling [15,16]. Muc5ac, one of the genes responsible for mucin production, is a major component of the mucus layer that lines the airways in the respiratory system and plays a pivotal role in airway defense and mucus clearance, contributing to the regulation of lung inflammation [17,18]. Mmp13 encodes a protease enzyme that contributes to tissue repair and remodeling under normal conditions but can also contribute to tissue damage and inflammation when dysregulated in lung diseases [19,20]. In our study, PM10 induced the mRNA levels of Fizz1, Mmp13, and Muc5ac more than 2-fold compared to the NTC group, while pretreatment with 11,17diHDoPE remarkably decreased the mRNA levels of all three genes (Figure 1c). These results suggest that pretreatment with 11,17diHDoPE alleviates the PM10-induced inflammatory responses in mouse lung tissue.

### 2.2. Untargeted Metabolite Screening Reveals 11,17diHDoPE Alters the Levels of Inflammation-Related Metabolites in PM10-Induced Inflamed Mouse Lung Tissue

**Figure 2:**
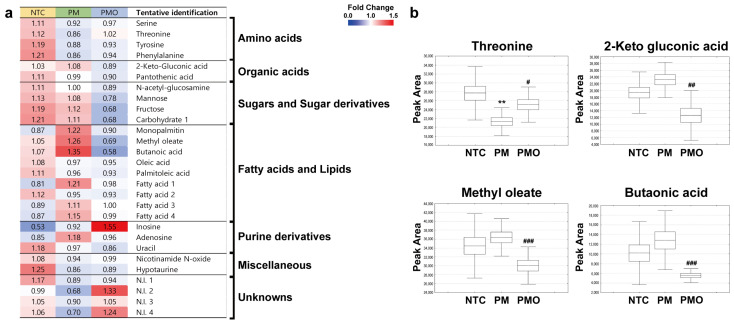
Comparison of metabolite profiles of mouse lung tissues by GC-TOF-MS analysis. (**a**) Heatmap shows metabolites identified in the lung tissues of three different groups of mice (NTC, *n* = 5; PM, *n* = 4; PMO, *n* = 4). Relative metabolite levels were normalized to the mean value and shown in color in each box. A total of 19 metabolites showed significantly different levels between NTC, PM, and PMO groups. Statistical significance was assessed using one-way analysis of variance (*p* < 0.05) and the PLS-DA model (VIP value > 0.7). (**b**) Box and whisker plots depicting the levels of key metabolites involved in inflammation in the lung tissue of the NTC, PM, and PMO mouse groups (NTC, *n* = 5; PM, *n* = 4; PMO, *n* = 4). Four key metabolites were selected based on two criteria. (1) Metabolite levels were altered after PM10 exposure and restored by 11,17diHDoPE treatment, and (2) Metabolite levels showed significant differences between the PM and PMO groups. Independent *t*-test was used to evaluate the statistical significance of the differences between NTC and PM groups (** *p* < 0.01) or between PM and PMO groups (# *p* < 0.05, ## *p* < 0.01, ### *p* < 0.001).

Additional experiments were conducted to investigate the metabolic impact of 11,17diHDoPE on inflamed mouse lung tissue. Gas chromatography time-of-flight mass spectrometry (GC-TOF-MS) analysis-based metabolite profiling was conducted using lung tissue obtained from NTC, PM, and PMO groups. Multivariate statistical analysis was initially carried out to assess the distinguishability of metabolite profiles among these three groups of mice (Figure S2). In principal component analysis score plots, metabolite profiles of NTC, PM, and PMO groups did not show clear separation. However, upon applying the partial least squares–discriminant analysis (PLS-DA) model using supervised methods, the three experimental groups were distinct from each other with PLS2 (4.25%) and PLS1 (6.20%) variables distinguishing PM10-treated groups (PM and PMO) from NTC. The quality of the PLS-DA model was assessed by R^2^X_(cum)_ = 0.250, R^2^Y_(cum)_ = 0.993, Q^2^_(cum)_ = 0.844 and cross-validation analysis (*p* = 7.31 × 10^−6^), indicating its validity. We then examined the metabolites that showed significantly different levels between the three groups of mice based on statistical analysis. For statistical analysis, variable importance in projection (VIP) values (>0.7) and one-way analysis of variance (ANOVA; *p* < 0.05) were used. As a result, we identified a total of 19 metabolites that exhibited significantly different levels between NTC, PM, and PMO groups. These are four amino acids, two organic acids, five fatty acids and lipids, three carbohydrates, three purines, and two miscellaneous compounds, showing significant differences among the three groups based on the statistical criteria (Figure 2a, Table S1). The significance was confirmed using the VIP value and one-way ANOVA *p*-value of discriminated metabolites. Additionally, the relative peak area was normalized to the mean value and visualized on a heatmap. Among these metabolites, four metabolites, threonine, 2-keto gluconic acid, butanoic acid, and methyl oleate, showed significantly different levels between the PM and PMO groups (Figure 2b). Threonine is known as a modulator of inflammatory and anti-inflammatory cytokine release [21,22]. 2-keto gluconic acid, butanoic acid, and methyl oleate have been reported to be involved in regulating inflammatory response [23,24,25,26]. Metabolite profiling results showed that threonine levels were significantly reduced in the PM group compared to NTC, whereas they recovered by more than 1.18-fold in the PMO group. Conversely, the levels of three metabolites, 2-keto gluconic acid, butanoic acid, and methyl oleate, increased after PM10 exposure but significantly decreased by 0.83-fold, 0.43-fold, and 0.54-fold, respectively, in the PMO group. In summary, PM10-induced metabolic changes were identified in mouse lung tissues using untargeted metabolite profiling. Notably, 11,17diHDoPE altered the levels of four metabolites associated with inflammation: threonine, 2-keto gluconic acid, butanoic acid, and methyl oleate.

### 2.3. Serum Oxylipin Profiling Unveils the Anti-Inflammatory Effects of 11,17diHDoPE in Mice Exposed to PM10

**Figure 3:**
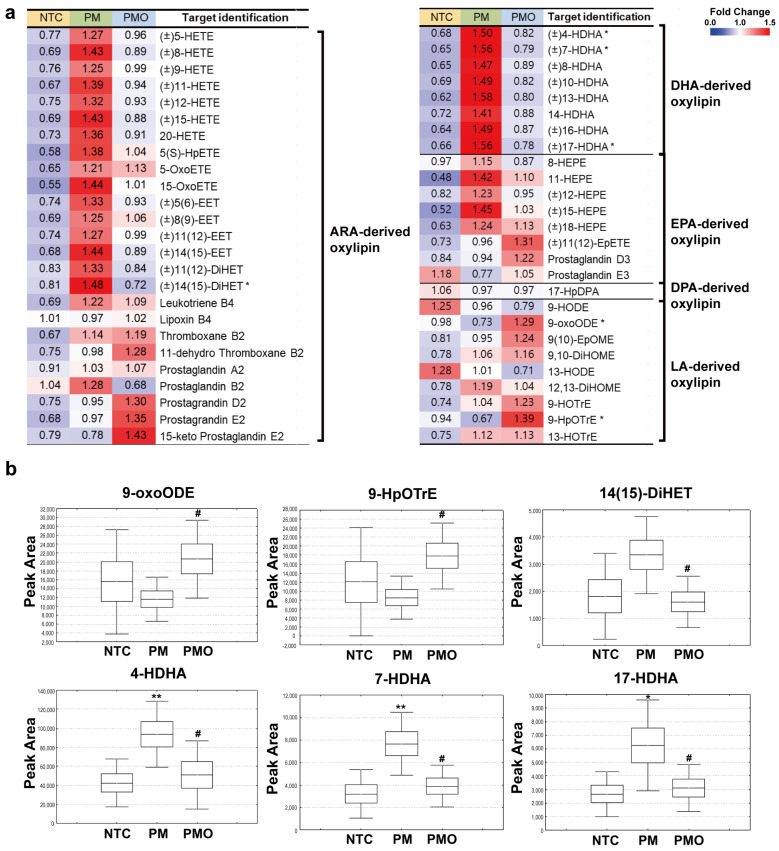
Oxylipin profiling in mouse serum by LC-triple-quadrupole-MS analysis. (**a**) Heatmap depicts oxylipin levels identified in the serum in NTC, PM, and PMO groups (NTC, *n* = 7; PM, *n* = 7; PMO, *n* = 7). Relative oxylipin levels were normalized to the mean value and shown in color in each box. Oxylipins showing statistical significance among the three groups are indicated with an asterisk (* p < 0.05). One-way analysis of variance was conducted to assess statistical significance. (**b**) Box and whisker plots show key oxylipin levels associated with inflammation measured in the serum of NTC, PM, and PMO mouse groups (NTC, *n* = 7; PM, *n* = 7; PMO, *n* = 7). Six oxylipins were selected based on two criteria. (1) Metabolite levels changed in PM and recovered in PMO, and (2) metabolite levels show significant differences between PM and PMO groups. Independent *t*-test was used to assess the statistical significance of the differences between NTC and PM groups (* *p* < 0.05, ** *p* < 0.01) between PM and PMO groups (# *p* < 0.05). NTC, non-treated control; PM, PM10 injected group; PMO, PM10, and 11,17diHDoPE co-injected group.

We conducted a detailed analysis of oxylipin levels in serum obtained from NTC, PM, and PMO mouse groups, detecting a total of 51 oxylipins by LC-triple-quadrupole-MS analysis (Table S2). These include 25 arachidonic acid-, eight docosahexaenoic acid-, eight eicosapentaenoic acid-, one DPA-, and nine linoleic acid-derived oxylipins (Figure 3a). Among them, six oxylipins regulated by 11,17diHDoPE and involved in inflammatory responses were visualized in box and whisker plots (Figure 3b). Notably, octadecadienoic acid (9-oxoODE) and hydroperoxy-octadecatrienoic acid (9-HpOTrE), known immunomodulators associated with the macrophage pathway [27,28], significantly increased 1.77- and 2.09-fold in the PMO group compared to the PM group. In contrast, dihydroxy-eicosatrienoic acid((±)14(15)-diHET), hydroxy-docosahexaenoic acid, ((±)4-HDHA), (±)7-HDHA, and (±)17-HDHA) significantly decreased by 0.48-, 0.54-, 0.51-, and 0.50-fold, respectively, in the PMO group compared to the PM group. These four oxylipins are known to be associated with inflammatory responses, particularly (±)7-HDHA and (±)17-HDHA, which have been reported to promote the polarization of M2 macrophages [29,30,31]. These results suggest that pretreatment with 11,17diHDoPE upregulates the linoleic acid-derived oxylipin pathway and downregulates arachidonic acid- and docosahexaenoic acid-derived oxylipin pathways in mouse serum. As these oxylipins are known to be produced by the LOX and CYP enzyme families, we also assessed the expression levels of relevant genes (Figure S3). The expression level of Alox5 and Alox12, LOX family-related genes, showed no significant differences between the NTC, PM, and PMO groups. However, the expression levels of CYP family-related genes, Cyp4f13 and Cyp4f39, were significantly increased in the PM group but decreased in the PMO group. These findings suggest that PM10 affects the levels of specific oxylipin subsets in mouse serum by regulating genes related to the CYP family. Likewise, 11,17diHDoPE can modulate the levels of inflammation-related oxylipins by regulating gene expression in oxylipin production.

### 2.4. Correlation of Metabolic and Gene Expression Changes Implicates the Role of 11,17diHDoPE on Macrophage Activity

**Figure 4:**
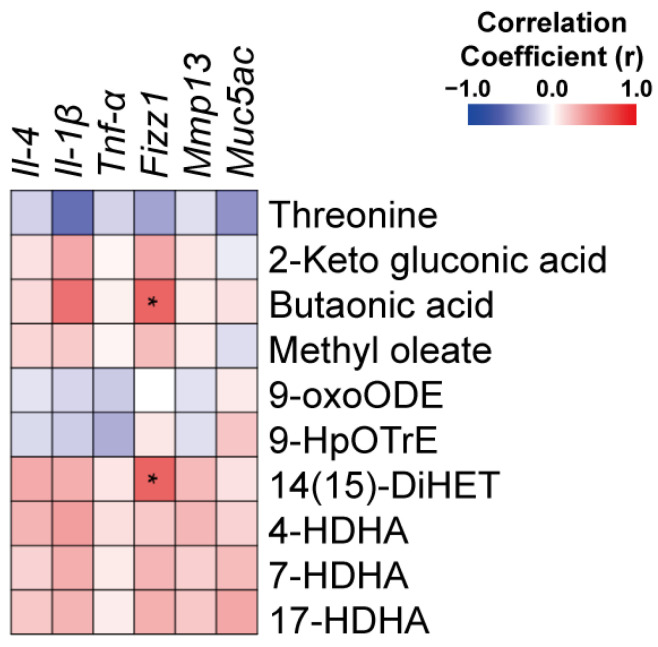
Correlation analysis between key metabolites levels and inflammatory gene expression levels. A correlation study between the levels of selected metabolites and inflammation-related gene expression was performed in a group of mice administered PM10 alone and a group of mice co-injected with PM10 and 11,17diHDoPE. Positive correlations are shown in red, and negative correlations in blue. An asterisk (*) denotes statistical significance, with * *p* < 0.05.

Based on observing potential associations between gene expression and metabolite levels, we conducted a correlation analysis between inflammation-related genes and metabolites (Figure 4 and Figure S4). Compared to the PM group, certain metabolites such as threonine, 9-oxoODE, and 9-HpOTrE were found at higher levels in the PMO group. Notably, these increased metabolites generally showed a negative correlation with the expression of genes that cause inflammation. These inflammatory genes include pro-inflammatory cytokine genes IL-4, IL-1b, TNF-α and lung function-related genes Fizz1, Mmp13, and Muc5ac. Conversely, metabolites such as 2-ketogluconic acid, butanoic acid, methyl oleate, (±)14(15)-diHET, (±)4-HDHA, (±)7-HDHA, and (±)17-HDHA were found at lower levels in the PMO group compared to the PM group. This decrease generally correlated positively with the expression of genes involved in inflammation. Particularly noteworthy, statistically significant positive correlations were observed between butanoic acid and Fizz1 expression (r = 0.59) and between 14(15)-DiHET and Fizz1 expression (r = 0.60). While existing studies lack clear evidence of 14(15)-DiHET’s involvement in inflammatory responses, butanoic acid is well recognized for its role in promoting M2 macrophage polarization [32]. The Fizz1 is also known to be secreted from M2 macrophages [15]. From the correlation analysis between metabolite alterations and gene expression changes, we have identified significant associations between specific metabolites and the Fizz1 gene. These findings further implicate a potential role for 11,17diHDoPE in interfering with the differentiation of M2 macrophages, potentially resulting in a decrease in Fizz1 secretion.

### 2.5. Pretreatment with 11,17diHDoPE Inhibits the Macrophage Differentiation in Inflamed Mouse Lung Tissue

**Figure 5:**
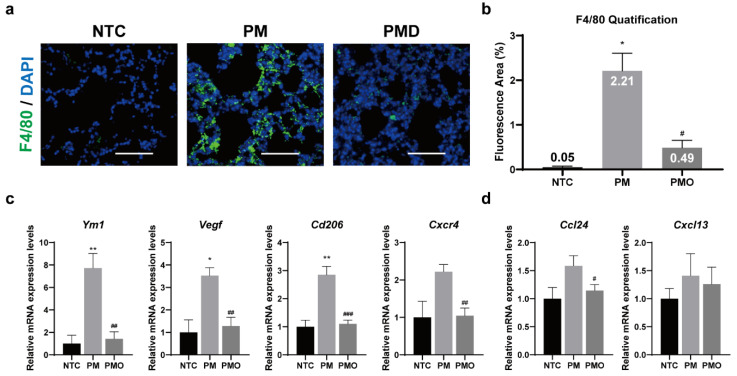
Effect of 11,17diHDoPE on macrophage recruitment in mouse lung tissue with PM10-induced inflammation. (**a**) Immunohistochemical analysis of macrophage recruitment in mouse lung tissue (NTC, *n* = 4; PM, *n* = 4; PMO, *n* = 4). The fluorescence of the macrophage detection marker F4/80 is shown in green. Cell nuclei are counterstained with DAPI and appear blue. Images were taken at 200× magnification and captured with Nikon Image software (version 4.60). The scale bar represents 200 μm. (**b**) Quantification of F4/80 positive fluorescent areas in five independent immunohistochemical images. (**c**) Relative mRNA levels of genes associated with anti-inflammatory M2 macrophage in mouse lung tissue (NTC, *n* = 7; PM, *n* = 7; PMO, *n* = 7). mRNA levels of genes related to M2 macrophage were normalized to Gapdh mRNA levels. (**d**) Relative mRNA levels of chemokines released from M2 macrophage in mouse lung tissue. Results are shown as mean ± standard error of the mean. The Mann–Whitney test was used to assess the statistical significance of the differences between WT and PM (* *p* < 0.05, ** *p* < 0.01) and between PM and PMO (# *p* < 0.05, ## *p* < 0.01, ### *p* < 0.001). NTC, non-treated control; PM, PM10 injected group; PMO, PM10, and 11,17diHDoPE co-injected group.

To further validate the anti-inflammatory effects of 11,17diHDoPE by regulating the differentiation of M2 macrophages, we investigated the role of 11,17diHDoPE on macrophage activity in mouse lung tissue with PM10-induced inflammation. The localized accumulation of macrophages is a key indicator of an inflammatory response [33]. To determine whether 11,17diHDoPE could prevent the recruitment of macrophages at sites of inflammation, mouse lung tissues were immuno-stained with F4/80, a fluorescent macrophage detection marker. Immunohistochemical analysis of lung tissues obtained from the PM group clearly showed higher amounts of immunofluorescent areas (Figure 5a). However, only a small amount of fluorescent area was detected in the lung tissues of the PMO group, and it was rarely observed in the NTC group. The fluorescent areas were further quantified in five independent immunohistochemical images obtained from each group. The PM group showed an approximately 44-fold increase in fluorescent areas in lung tissues compared to the NTC group. Conversely, the PMO group exhibited only a 10-fold increase compared to the NTC group, representing a 5-fold decrease compared to the PM group (Figure 5b). In addition, histological examination using hematoxylin and eosin (H&E) staining revealed a remarkable increase in immune cell infiltration around alveoli in the PM group. In contrast, immune cell infiltration was attenuated in the PMO group, resembling that of the NTC group (Figure S5). These findings indicate that PM10 induces macrophage recruitment and immune cell infiltration at the inflamed region, while 11,17diHDoPE interferes with macrophage accumulation and attenuates immune cell infiltration. We further assessed the expression levels of genes associated with M2 macrophages, including Ym1, Vegf, Cd206, and Cxcr4 genes [34,35]. Upon PM10 exposure, the mRNA levels of Ym1, Vegf, Cd206, and Cxcr4 genes were highly increased up to 8-fold compared to the NTC group (Figure 5c). However, the mRNA levels of these genes were significantly reduced to the NTC levels by pretreatment with 11,17diHDoPE. In addition, upon PM10 stimulation, the mRNA levels of chemokines Ccl24 and Cxcl13 released from macrophages [36,37] increased by 1.5-fold (Figure 5d). Conversely, pretreatment with 11,17diHDoPE led to a decrease in the mRNA levels of these chemokines. Taken together, these results indicate that 11,17diHDoPE may exert anti-inflammatory effects on PM10-induced lung inflammation by preventing the differentiation of macrophages at the site of inflammation.

## 3. Discussion

The ongoing industrialization has led to a significant escalation in air pollution, posing a substantial threat to global health. Among air pollutants, PM stands out as a prominent concern. Large particles are generally filtered by cilia and mucus in the nose and throat, whereas particles smaller than about 10 μm can penetrate deeper into the lungs, including the bronchioles and alveoli. Therefore, long-term inhalation of PM10 can induce severe inflammatory responses, which lead to various respiratory diseases, such as asthma and chronic obstructive pulmonary disease (COPD) [38]. It can also induce skin diseases, heart diseases, psychological disorders, and premature death [39,40,41,42]. As PM10-induced health problems increase worldwide, the International Agency for Research on Cancer (IARC) classified airborne particulate matter as a Group 1 carcinogen [43]. However, the detailed underlying mechanisms of how PM10 induces inflammation are not fully understood, and there are no commercially available therapeutic agents for treating PM10-induced inflammation to date.

In this study, we demonstrated the anti-inflammatory effects of the SPM isomer 11,17diHDoPE via the integrative analysis of gene expression and metabolic changes using a mouse inflammation model. Intranasal exposure to the air pollutant PM10 in mice clearly induced inflammatory responses in lung tissue. However, pretreatment with 11,17diHDoPE significantly reduced the expression levels of pro-inflammatory cytokines IL-4 and IL-1β, as well as a gene associated with pulmonary inflammation. Untargeted metabolite screening identified a total of 19 metabolites whose levels changed after PM10 exposure in mouse lung tissues and were restored by 11,17diHDoPE treatment. Among them, four metabolites: threonine, 2-keto gluconic acid, butanoic acid, and methyl oleate, are known to function as modulators of inflammation. Specifically, threonine deficiency promotes the secretion of inflammatory cytokines and inhibits anti-inflammatory cytokines. Butanoic acid promotes the polarization of M2 macrophages [23,24,32]. Further comprehensive oxylipin profiling in mouse serum identified 51 endogenous oxylipins whose levels were changed upon PM10 exposure but recovered with 11,17diHDoPE treatment. Notably, six oxylipins, 9-oxoODE, 9-HpOTrE, (±)4-HDHA, (±)7-HDHA, and (±)17-HDHA, are known to be involved in regulating inflammatory responses [27,28,29,30,31]. While 9-oxoODE and 9-HpOTrE induce anti-inflammatory effects by reducing macrophage activity, (±)17-HDHA promotes polarization of M2 macrophages. While our study identified a limited number of metabolites and oxylipins as regulators of inflammation based on existing literature, additional studies may deepen our understanding of the comprehensive metabolite profiles associated with the anti-inflammatory effects of 11,17diHDoPE.

Using an integrative analysis of metabolite and gene expression changes, we have demonstrated that changes in endogenous oxylipin levels following 11,17diHDoPE treatment are predominantly regulated by the CYP enzyme family. Additional correlation analysis between metabolite alteration and gene expression changes revealed a significant positive relationship between butanoic acid and Fizz1 upon 11,17diHDoPE treatment. Notably, butanoic acid is well recognized for promoting the differentiation of M2 macrophages, and Fizz1 is known to be released from M2 macrophages [15,32,34]. The immunohistochemical and gene expression analysis results further verified that 11,17diHDoPE treatment could inhibit the recruitment of M2 macrophages at the site of inflammation. These findings suggest that the anti-inflammatory effects of 11,17diHDoPE are mainly achieved by modulating macrophage activity. Macrophages are essential in regulating inflammation and are divided into two subtypes, M1 and M2 [44]. M1 macrophages initiate pro-inflammatory responses in the early stages of inflammation, whereas M2 macrophages contribute to wound healing and tissue repair later. The anti-inflammatory effect of 11,17diHDoPE demonstrated in this study primarily targets M2 macrophages, but its influence is not limited to M2 macrophages and may also extend to suppressing M1 macrophage activity. Accordingly, 11,17diHDoPE exerts its anti-inflammatory effect by modulating the macrophage pathway. Additional studies may be needed to uncover the precise mechanism of how 11,17diHDoPE modulates macrophage activity to alleviate inflammation.

In summary, we provided compelling evidence of the anti-inflammatory effects of 11,17diHDoPE in PM10-induced mouse lung inflammation. Our findings provided fundamental information to understand the mechanism of PM10-induced lung inflammation and how 11,17diHDoPE can alleviate this lung damage by regulating inflammation-related gene expression and metabolite levels. This in vivo study will further contribute to the design strategy for applying 11,17diHDoPE as a therapeutic agent for treating PM10-induced respiratory diseases. Although the anti-inflammatory effects of pro-resolution mediators have been mostly demonstrated using in vitro cell culture models, a limited number of studies demonstrated the pro-resolution activities of oxylipins using mouse models. One of the SPMs, resolvin E1 (RvE1), is known to alleviate asthmatic inflammation by suppressing the production of pro-inflammatory cytokine IL-23, which results in the modulation of the T cell population (Figure S6a). Treatment of another resolvin subtype RvD1 to cigarette smoke-induced COPD mice increased the levels of anti-inflammatory cytokine IL-10 and promoted the differentiation of M2 macrophages to clear neutrophil accumulation [45]. Pretreatment with synthetic maresin 1 (MaR1) reduced neutrophil infiltration in mice with zymosan-induced acute peritonitis [46]. Collectively, individual pro-resolving mediators appear to regulate different inflammation pathways, which is also distinct from 11,17diHDoPE. As a comparative study, we also tested the effect of different pro-resolving mediators, 7S MaR1 and 7S MaR1 n-3, in PM10-exposed mice (Figure S6b). While mice pretreated with 11,17diHDoPE did not show any mortality, administering either 7S MaR1 or 7S MaR1 n-3 in mice resulted in a 60% survival rate, indicating potential toxic effects of these mediators in mice. While the detailed mechanisms of how PM10 induces inflammation in respiratory epithelial cells are not fully understood, one best-described mechanism involves the IL-8 pathway [38,47]. Several pieces of evidence revealed that PM10 activates the Toll-like receptor-4 signaling pathway, leading to increased levels of pro-inflammatory cytokines, such as IL-8, which attract neutrophils to the inflamed region. Additionally, PM10 can generate reactive oxygen species, further increasing IL-8 levels and promoting neutrophil accumulation. Since the IL-8 gene is absent in the mouse genome, we were not able to test the effect of 11,17diHDoPE on the IL-8 pathway, further studies using human cell lines, such as PM10-induced asthma or COPD cell lines, could be beneficial for understanding the role of 11,17diHDoPE in the PM10-induced IL-8 pathway. During the activation of inflammatory responses, the human body secretes the natural metabolites, specifically pro-resolving mediators, to promote the resolution of inflammation. Indeed, clinical studies have revealed that the levels of the pro-resolving mediator lipoxin are decreased in severe asthma patients [48]. Although additional studies are needed to investigate whether the levels of 11,17diHDoPE are decreased in patients with PM10-induced chronic respiratory disorders, such as asthma and COPD, 11,17diHDoPE still holds potential as a significant biomarker for diagnosing PM10-induced respiratory diseases. Several pieces of evidence showed that one SPMs resolvin exhibited pro-resolution activity on asthma and COPD models [45,49]. Our study provided compelling in vivo evidence supporting the anti-inflammatory activity of exogenous 11,17diHDoPE treatment in PM10-exposed mice with acute inflammation. While additional studies are needed to investigate the long-term effect of exogenous 11,17diHDoPE treatment in chronic respiratory disease models, 11,17diHDoPE could serve as a potential therapeutic agent for the treatment of PM10-induced respiratory diseases.

## 4. Materials and Methods

### 4.1. 11,17diHDoPE Preparation

To prepare 11,17diHDoPE, the reaction was performed at 30 °C in a 50 mM HEPPS buffer (pH 8.5) containing 2 mM DPA as a substrate, 200 mM cysteine, and 0.5 mg/mL of arachidonate 9S-lipoxygenase from *Sphingopyxis macrogoltabida* with shaking at 200 rpm for 1 h. The reaction solution was extracted by adding an equal volume of ethyl acetate; the ethyl acetate layer was collected and dried by evaporation. The dried residue was dissolved in methanol and applied to a Prep-HPLC (Agilent 1260; Agilent, Santa Clara, CA, USA) equipped with a Nucleosil C18 column (10 × 250 mm; Phenomenex, Torrance, CA, USA), which was eluted at 30 °C at a flow rate of 6 mL/min. Product fraction was collected based on the peak observed at 202 nm. The collected product was dried by evaporation, and the dried product was used as 11,17diHDoPE.

### 4.2. PM10 Preparation

Particulate matter 10 (PM10, #ERM-CZ100, Saint Louis, MO, USA) was dissolved in PBS at 50 mg/mL concentration and used as the stimulant of mouse lung inflammation. To ensure the homogeneity of the PM10 solution, sonication was performed for 60 min using a sonicator (Sonics and Materials, Inc., Newtown, CT, USA).

### 4.3. Mice

Four-week-old female Balb/c mice were purchased from Orient Bio (Seongnam, Republic of Korea) and maintained under specific pathogen-free conditions with a 12 h light/dark cycle at room temperature and 30–70% relative humidity. Following habituation, 5-week-old mice were divided into three groups to determine the effect of 11,17diHDoPE on PM10-induced mouse lung inflammation. 11,17diHDoPE (2 mg/kg) was pretreated via the intranasal route on mice, and PM10 (300 μg) was intranasally injected after 5 min. After the resting period, the second injection was performed with the same condition on day 5 and named the PMO group. The PM group was administered with the same volume of PBS instead of 11,17diHDoPE, and the NTC group was administered with the same amount of PBS via the intranasal route instead of 11,17diHDoPE and PM10. The following day, all mice were euthanized by CO_2_ inhalation, and lung tissue and serum were collected to investigate the impacts of 11,17diHDoPE on PM10-induced pulmonary inflammation. All animal procedures were approved by the Institutional Animal Care and Use Committee of Konkuk University (Seoul, Republic of Korea).

### 4.4. RT-qPCR

According to the manufacturer’s instructions, RNA was extracted from mouse lung tissue using the PureLink RNA Mini Kit (12183018A, Invitrogen, Carlsbad, CA, USA). cDNA was synthesized from total RNA by reverse transcription using SuperScript III Reverse Transcriptase (18080-400, Invitrogen). qPCR was then performed on a LightCycler 96 Instrument (#05815916001, Roche, Basel, Switzerland) using the SsoAdvanced Universal SYBR Green Supermix (#1725271, BioRad, Hercules, CA, USA) and gene-specific primers. Sequences of primers used for RT-qPCR are listed in Table S3. All mRNA levels were normalized to Gapdh levels.

### 4.5. Sample Preparation for Metabolomic Studies

Metabolites were extracted from an average of 56.67 ± 8.94 mg of lung tissue (NTC, *n* = 5; PM, *n* = 4; PMO, *n* = 4) and 70 µL of serum (NTC, *n* = 7; PM, *n* = 7; PMO, *n* = 7). Following this, 1 mL of methanol containing 1 mg of an internal 2-chlorophenylalanine standard was added to both lung tissue and serum samples, followed by homogenization using a mixer mill and sonicator for 10 min each. After homogenization, the suspensions were held at −20 °C for 60 min and then centrifuged at 17,000× *g* and 4 °C for 10 min. The supernatants were filtered through a 0.2 μm polytetrafluoroethylene filter and dried using a speed vacuum concentrator, Modulspin 31 (Biotron, Wonju, Republic of Korea). For GC–TOF–MS analysis, dried samples were oximated with 50 µL of methoxyamine hydrochloride (20 mg/mL in pyridine) for 90 min at 30 °C and silylated with 50 µL of N-methyl-N-(trimethylsilyl) trifluoroacetamide for 30 min at 37 °C. For liquid chromatography (LC)-Triple-Quadrupole-MS analysis, the extracts were reconstituted in methanol (5 mg/mL), filtered, and then subjected to analysis.

### 4.6. GC-TOF-MS Analysis

GC-TOF–MS analysis was performed using an Agilent 7890 gas chromatography system (Agilent Technologies, Palo Alto, CA, USA) coupled with an L-PAL3 auto-sampler (LECO Corp., St. Joseph, MI, USA) and equipped with a Pegasus^®^ HT TOF MS system. An Rtx-5MS column (i.d., 30 m × 0.25 mm, 0.25 µm particle size; Restek Corp., Bellefonte, PA, USA) was used with a constant flow of 1.5 mL/min of helium as the carrier gas. Samples (1-µL aliquots) were injected into the GC with splitless mode. The oven temperature was maintained at 75 °C for 2 min, then incrementally raised 15 °C/min to 300 °C, and finally held for 3 min. The front inlet and transfer line temperatures were 250 and 240 °C, respectively. The electron ionization was carried out at −70 eV, and full scanning over the 50–700 *m*/*z* range was used for mass data collection. The GC–TOF–MS data were acquired and preprocessed using the LECO Chroma TOF™ software (version 4.44, LECO Corp., St. Joseph, MI, USA) and converted into the NetCDF format (*.cdf) using the LECO Chroma TOF™ software. After conversion, peak detection, retention time correction, and alignment were processed using the Metalign software package “https://www.metalign.nl (accessed on 13 May 2024)”. The box and whisker plots were rendered using the relative peak area of unique masses of metabolites by STATISTICA 7 software (StatSoft Inc., Tulsa, OK, USA). Heat maps were rendered with the fold-change of each metabolite normalized to the mean value, using the relative peak area integrated by Metalign. The correlation map was also obtained using the PASW Statistics software (version 18.0.0; SPSS Inc., Chicago, IL, USA).

### 4.7. LC-Triple-Quadrupole-MS Analysis

LC-triple-quadrupole-MS analysis was conducted using a Nexera2 LC system (Shimadzu Corp., Kyoto, Japan) coupled with a triple-quadrupole MS equipped with an electrospray source (LC-MS 8040, Shimadzu). Five microliters were injected into a Syncronis C18 column (100 × 2.1 mm, 1.7 µm, Thermo Scientific, Waltham, MA, USA) with a mobile phase consisting of 0.1% *v*/*v* formic acid in water (solvent A) and 0.1% *v/v* formic acid in acetonitrile (solvent B) at a flow rate of 300 µL/min. The gradient was set at 15% solvent B for 1 min and linearly increased from 15–100% over 12 min. The total run time, including re-equilibration of the column to the initial conditions, was 15 min. Operating conditions for the MS platform were: capillary voltage −3000 V, capillary temperature 350 °C, vaporizer temperature 300 °C, sheath gas 3 L/min, ion sweep gas 2.0 Arb, Aux gas 10 Arb, and drying gas 8 L/min. The subsequent multiple reaction monitoring transitions used are summarized in Table S1. Relative peak areas of each oxylipin were collected by the Labsolutions program (version 5.85, Shimadzu Corporation, Kyoto, Japan). Data visualization and correlation analysis for LC-Triple-Quadrupole-MS followed the same procedures described for GC-TOF-MS analysis.

### 4.8. Histological Analysis

Mouse lung tissues were fixed in 10% formalin, dehydrated in an ethanol series and xylene, and embedded in paraffin according to standard protocols. Paraffin blocks were sectioned at 4 μm thickness. For immunohistochemistry, antigen unmasking was performed in a TintoRetriever Heat Retrieval System (Bio SB Inc., Goleta, CA, USA) using 10 mM sodium citrate buffer (pH 6.0) for 10 min. Sections were blocked with 3% normal goat serum at room temperature for 1 h. Tissues were then incubated overnight at 4 °C with 5 μg of anti-F4/80 (#sc-377009, Santa Cruz, CA, USA). After washing with PBS, tissues were incubated with Alexa Fluor 488-conjugated anti-mouse IgG (#R37120; Invitrogen) for 1 h at room temperature. Slides were mounted with Prolong Gold Antifade Mountant with DAPI (P36931, Invitrogen), imaged under a Nikon Eclipse Ts2R (Nikon, Tokyo, Japan), and analyzed using the Nikon image browser. The fluorescent areas were quantified using ImageJ software (version 1.8.0).

### 4.9. Statistics and Reproducibility

RT-qPCR results were presented as means ± standard error of the mean, and statistical significance was analyzed by unpaired *t*-test (Mann–Whitney test) using GraphPad Prism 9.4.0. (GraphPad Software, San Diego, CA, USA). ANOVA was conducted to assess the statistical significance among the three groups in the heat maps. Duncan’s multiple range tests were used to determine the significance of ANOVA. In the box and whisker plot, the significance of the comparisons between the two groups of NTC and PM, as well as between PM and PMO, was evaluated using Student’s *t*-test. The significance of the correlation analysis was determined by the Pearson correlation coefficient significance test, and these statistical analyses were performed using PASW Statistics software (version 18.0.0). Multivariate statistical analysis employed SIMCA-P+ (version 12.0; Umetrics, Umeå, Sweden). Principal component analysis and PLS-DA were conducted to visualize the metabolite differences among each group. Additionally, they were utilized for variable selection, with a threshold of variable importance projection value > 0.7 applied in the PLS-DA model. The PLS-DA model was evaluated using cumulative R2X, R2Y, and Q2 values calculated using single cross-validation. The *p*-value was obtained from cross-validation ANOVA of PLS-DA.

## Data Availability

Additional supporting data related to this study are available from the corresponding author upon reasonable request. Source data for metabolite profiling and RT-qPCR are presented in Supplementary Tables S1–S3.

## Supplementary Materials

The following supporting information can be downloaded at: https://www.mdpi.com/article/10.3390/ijms25105360/s1.
