# Supplementary material for: Gene Expression and Metabolome Analysis Reveals Anti-Inflammatory Impacts of 11,17diHDoPE on PM10-Induced Mouse Lung Inflammation
Source: Int J Mol Sci. 2024 May 14;25(10):5360. doi: 10.3390/ijms25105360 (PMC11121355; doi:10.3390/ijms25105360)
Supplement: Supplementary file 1 [file ijms-25-05360-s001.zip › ijms-2989765-supplementary.pdf]

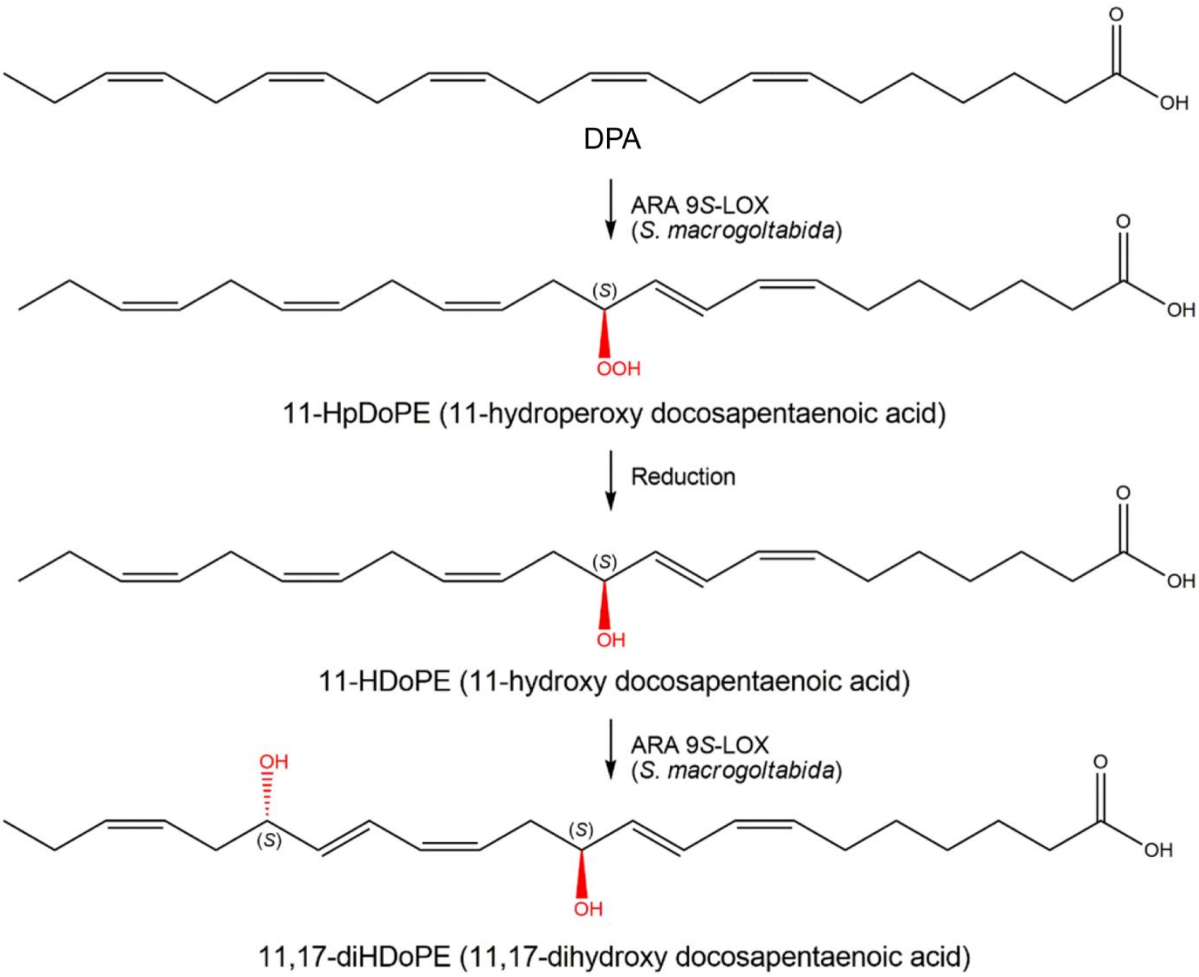

Figure S1. Biosynthetic pathway of DPA to 11,17diHDoPE by ARA 9S-LOX of *S. macrogoltabida.* ARA, arachidonate.

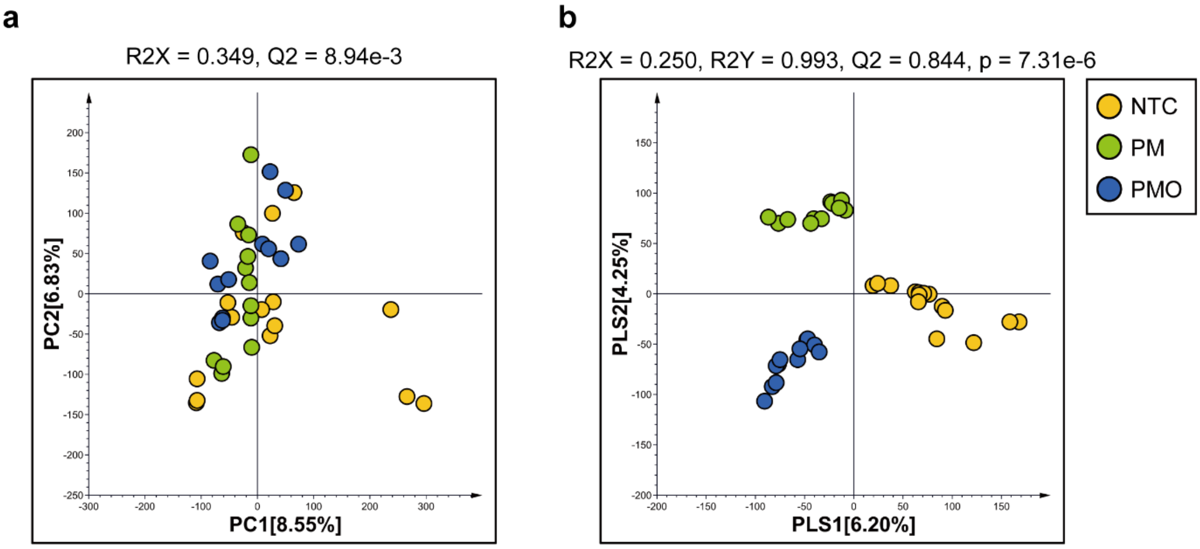

Figure S2. Multivariate analysis in lung tissue using GC-TOF-MS. (a) Principal component analysis and (b) PLS-DA score plots for mouse lung tissue (NTC, n = 5; PM, n = 4; PMO, n = 4). All analyses were performed in three independent replicates. PLS-DA, partial least squares discriminant analysis; PM, particulate matter; NTC, non-treated control.

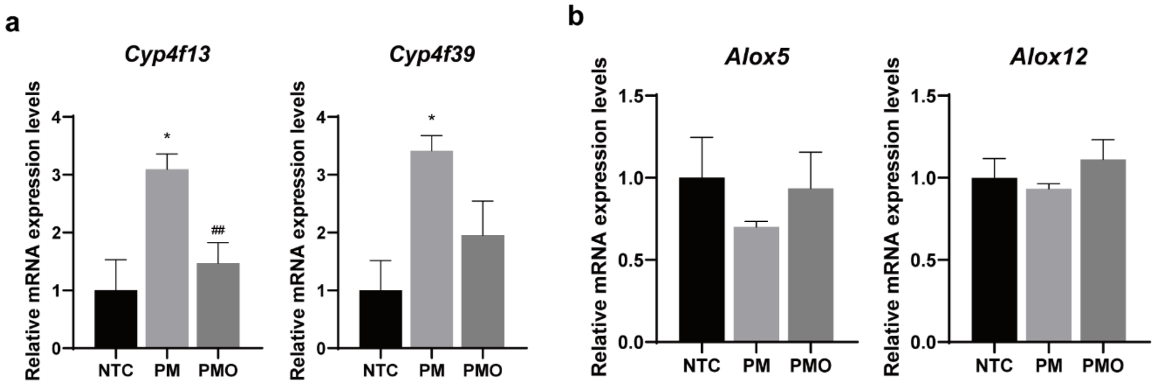

Figure S3. Effect of 11,17diHDoPE on the expression of genes associated with oxylipin-metabolic enzymes in mice exposed to PM10. (**a**) Relative mRNA levels of genes associated with the cytochrome p450 enzyme family in lung tissues from NTC, PM, and PMO mouse groups (NTC, n = 7; PM, n = 7; PMO, n = 7). mRNA levels of cytochrome p450 family genes were normalized to Gapdh mRNA levels. Results are shown as mean ± standard error of the mean. An unpaired t-test (Mann-Whitney test) was used to assess the statistical significance of the differences between NTC and PM (*p <0.05) and between PM and PMO (##p <0.01). (**b**) Relative mRNA levels of genes associated with LOX enzyme family in lung tissues from NTC, PM, and PMO mice groups.

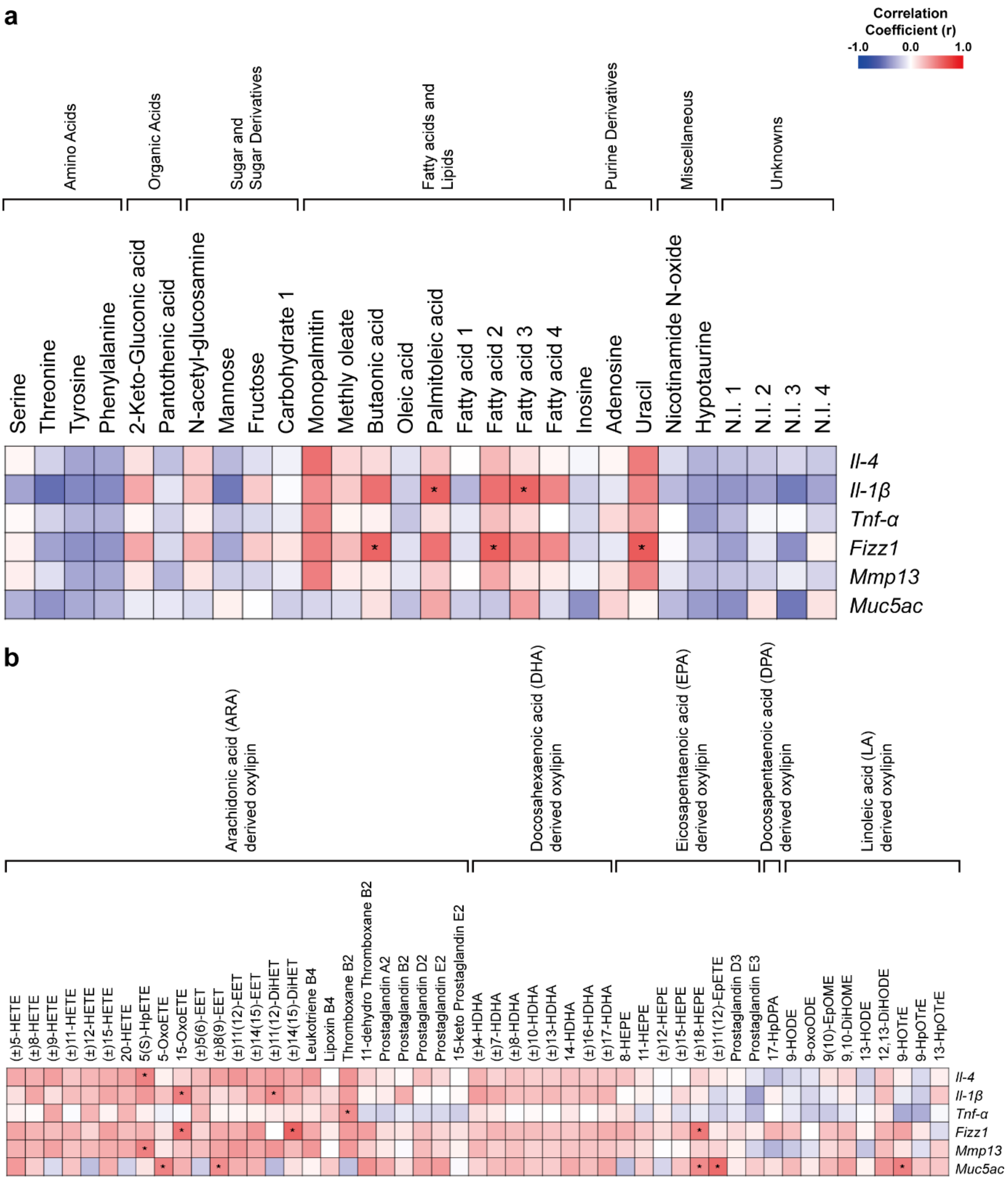

Figure S4. Correlation analysis between total metabolite levels and inflammation-related gene expression levels. (**a**) Correlation analysis between metabolite levels identified in lung tissues and inflammation-related gene expression levels in PM and PMO groups. (**b**) Correlation analysis between oxylipin levels identified in serum and inflammation-related gene expression levels in PM and PMO groups. Positive correlations are depicted in red, negative correlations in blue, and statistical significance is indicated by an asterisk (*p <0.05) where applicable.

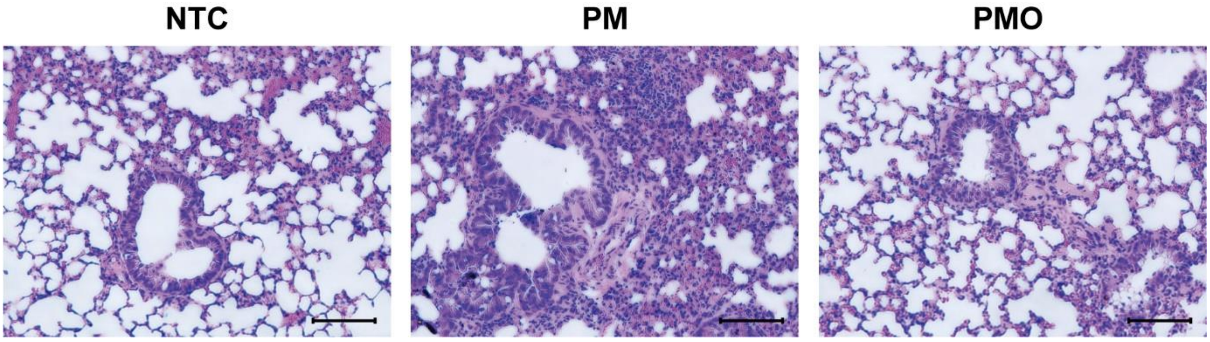

Figure S5. Histological assessment of immune cell infiltration at mouse lung tissues using hematoxylin and eosin (H&E) staining. Intense immune cell infiltration was induced by PM10 exposure in the PM group compared to NTC, whereas immune cell infiltration was markedly attenuated around alveoli in the PMO group. Images were taken at 100x magnification and captured with Nikon Image software. The scale bar represents 20 μm.

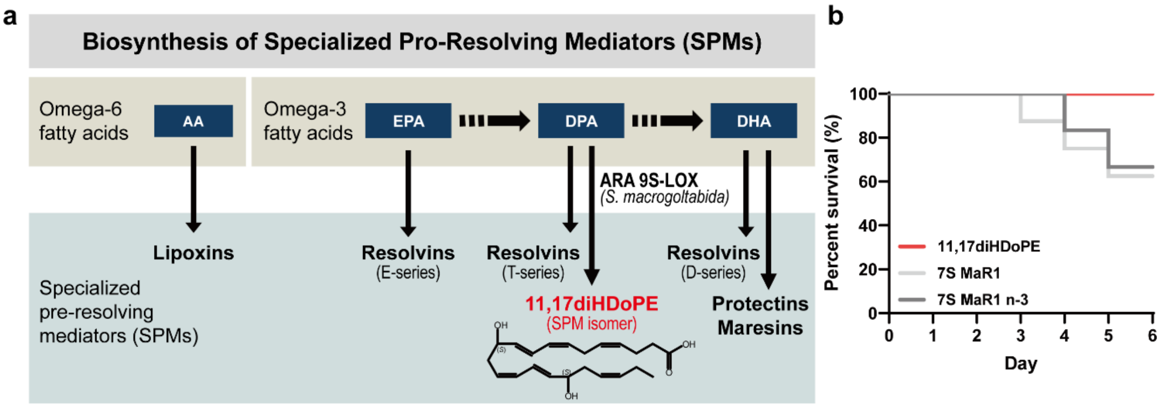

Figure S6. Biosynthesis of omega-6 and omega-3 fatty acid derived pro-resolving mediators (SPMs) with their effects in PM10-exposed mice. (a) A schematic of biosynthetic process of SPMs. 11,17diHDoPE was synthesized from DPA to 11,17diHDoPE by ARA 9S-LOX of *S. macrogoltabida***.** AA, arachidonic acid; EPA, eicosapentaenoic acid; DPA, docosapentaenoic acid; DHA, docosahexaenoic acid. (b) Comparison of survival rates between mouse groups treated with three different oxylipins (11,17diHDoPE, n = 9; 7S MaR1, n = 10; 7S MaR1 n-3, n = 7). 11,17diHDoPE, 11,17diHDoPE-treated group; 7S MaR1, 7S MaR1-treated group; 7S MaR1 n-3, 7S MaR1 n-3--treated group.

Table S1. Lung tissue metabolites discriminating experimental groups based on metabolite profiling analyzed by GC-TOF-MS

No.	Tentative Identification	GC-TOF-MS							
		VIP 1	VIP 2	Ret (min)	Unique mass(m/z)	MS fragment pattern(m/z)	TMS	ID	One way ANOVA (p-value)
Amino acids									
1	Serine	0.80	1.27	8.01	218	73, 204, 218, 75, 147, 205, 74, 116, 219, 206	3	Lib/STD	0.045
2	Threonine	0.23	1.86	8.26	57	73, 57, 117, 101, 219, 218, 147, 75, 100, 74	3	Lib/STD	0.005
3	Phenylalanine	1.19	2.33	10.26	218	73, 218, 192, 100, 147, 75, 74, 91, 219, 59	2	Lib/STD	0.000
4	Tyrosine	1.91	2.63	12.48	218	73, 218, 100, 219, 147, 74, 75, 179, 220, 280	3	Lib/STD	0.000
Organic acids									
5	2-Keto-Gluconic acid	1.92	1.99	10.49	204	73, 147, 204, 103, 117, 201, 74, 205, 217, 75	5	Lib/MS	0.025
6	Pantothenic acid	2.03	1.43	12.84	291	73, 103, 75, 117, 157, 55, 201, 291, 159, 147	3	Lib/MS	0.012
Sugars and sugar derivatives									
7	Fructose	3.26	2.47	12.10	307	73, 103, 217, 147, 74, 133, 307, 117, 89, 104	5	Lib/STD	0.000
8	Carbohydrate 1	3.35	2.48	12.17	307	73, 103, 217, 147, 74, 307, 89, 117, 75, 133	5	Lib/MS	0.000
9	Mannose	2.83	2.21	12.20	201	73, 147, 160, 205, 103, 129, 319, 117, 157, 74	5	Lib/STD	0.002
10	N-acetyl-glucosamine	2.55	1.78	13.48	202	73, 147, 129, 87, 103, 202, 205, 133, 117, 157	4	Lib/STD	0.011
Fatty acids and lipids									
11	Butanoic acid	2.30	1.80	6.09	233	147, 73, 75, 117, 116, 148, 59, 101, 191, 66	2	Lib/MS	0.007
12	Palmitoleic acid	1.65	1.45	12.94	311	75, 73, 117, 129, 55, 67, 96, 81, 84, 69	1	Lib/STD	0.019
13	Methyl oleate	2.16	2.04	13.42	98	55, 74, 69, 67, 87, 83, 84, 96, 81, 97	0	Lib/STD	0.001
14	Oleic acid	2.91	2.39	14.11	339	117, 75, 129, 73, 55, 96, 145, 84, 98, 81	1	Lib/STD	0.035
15	Fatty acid 1	0.23	1.80	15.11	329	59, 72, 55, 60, 69, 67, 57, 126, 112, 83	0	Lib/MS	0.001
16	Fatty acid 2	2.77	2.59	15.22	367	75, 73, 55, 67, 69, 74, 79, 81, 54, 61	2	Lib/MS	0.004
17	Fatty acid 3	0.70	1.68	15.95	218	73, 129, 147, 103, 75, 55, 218, 57, 131, 203	2	Lib/MS	0.005
18	Fatty acid 4	0.20	1.34	15.96	270	73, 75, 79, 67, 91, 55, 117, 80, 129, 93	1	Lib/MS	0.004
19	Monopalmitin	0.39	1.46	16.13	371	73, 147, 57, 129, 55, 75, 103, 203, 71, 83	2	Lib/MS	0.042
Purines and pyrimidine									
20	Uracil	2.09	1.52	7.83	99	99, 73, 147, 241, 126, 113, 255, 256, 100, 131	2	Lib/STD	0.007
21	Inosine	3.46	2.41	16.18	230	73, 217, 230, 147, 245, 103, 193, 74, 75, 281	4	Lib/STD	0.000
22	Adenosine	0.21	1.57	16.49	236	73, 236, 230, 75, 217, 71, 103, 57, 245, 192	4	Lib/STD	0.030
Others									

23	Nicotinamide N-oxide	0.31	1.08	9.09	179	75, 179, 136, 78, 51, 76, 105, 180, 77, 61	1	Lib/MS	0.035
24	Hypotaurine	1.98	2.21	10.06	188	73, 100, 188, 59, 147, 174, 86, 114, 75, 74	3	Lib/STD	0.000
Unknowns									
25	N.I. 1	0.23	1.80	15.11	329	59, 72, 55, 60, 69, 67, 57, 126, 112, 83	0	Lib/MS	0.001
26	N.I. 2	2.77	2.59	15.22	367	75, 73, 55, 67, 69, 74, 79, 81, 54, 61	2	Lib/MS	0.004
27	N.I. 3	0.70	1.68	15.95	218	73, 129, 147, 103, 75, 55, 218, 57, 131, 203	2	Lib/MS	0.005
28	N.I. 4	0.20	1.34	15.96	270	73, 75, 79, 67, 91, 55, 117, 80, 129, 93	1	Lib/MS	0.004

Table S2. Serum oxylipins identified in experimental groups based on lipid profiling analyzed by LC-Tripe-quadrupole-MS

LC-Triple-quadrupole-MS								
**No.**	**Compound name**	**Molecular formula**	**Ret (min)**	**Precursor ion(m/z)**	**Adduct**	**Product ion(m/z)**	**One way ANOVA (p-value)**	**t-test p-value (PM vs PMO)**
Arachidonic acid derived oxylipins								
1	(±)5-HETE	C20H32O3	11.63	319.00	[M–H]–	115.05	0.176	0.201
2	(±)8-HETE	C20H32O3	11.40	319.00	[M–H]–	155.15	0.031	0.062
3	(±)9-HETE	C20H32O3	11.45	319.00	[M–H]–	179.35	0.141	0.332
4	(±)11-HETE	C20H32O3	11.27	319.00	[M–H]–	167.20	0.060	0.153
5	(±)12-HETE	C20H32O3	11.44	319.00	[M–H]–	179.20	0.074	0.156
6	(±)15-HETE	C20H32O3	11.11	319.00	[M–H]–	219.20	0.049	0.072
7	20-HETE	C20H32O3	11.44	319.00	[M–H]–	301.30	0.049	0.108
8	5(S)-HpETE	C20H32O4	8.88	335.00	[M–H]–	59.10	0.061	0.327
9	5-OxoETE	C20H30O3	12.20	317.00	[M–H]–	203.25	0.104	0.758
10	15-OxoETE	C20H30O3	11.45	317.00	[M–H]–	139.20	0.062	0.292
11	(±)5(6)-EET	C20H32O3	11.44	319.00	[M–H]–	163.30	0.072	0.153
12	(±)8(9)-EET	C20H32O3	11.62	319.00	[M–H]–	301.25	0.135	0.502
13	(±)11(12)-EET	C20H32O3	11.63	319.00	[M–H]–	257.25	0.136	0.264
14	(±)14(15)-EET	C20H32O3	11.11	319.00	[M–H]–	219.20	0.040	0.068
15	(±)11(12)-DiHET	C20H34O4	9.96	337.00	[M–H]–	167.20	0.593	0.438
16	(±)14(15)-DiHET	C20H34O4	9.70	337.00	[M–H]–	207.20	0.055	0.020
17	Leukotriene B4	C20H32O4	9.23	335.00	[M–H]–	195.20	0.276	0.665
18	Lipoxin B4	C20H32O5	8.50	351.00	[M–H]–	211.25	0.957	0.795
19	Thromboxane B2	C20H34O6	6.34	369.00	[M–H]–	169.25	0.433	0.914
20	11-dehydro Thromboxane B2	C20H32O6	8.04	367.00	[M–H]–	305.25	0.315	0.343
21	Prostaglandin A2	C20H30O4	8.67	333.00	[M–H]–	271.30	0.823	0.854
22	Prostaglandin B2	C20H30O4	8.42	333.00	[M–H]–	175.25	0.316	0.066
23	Prostagrandin D2	C20H32O5	7.27	351.00	[M–H]–	271.30	0.186	0.180
24	Prostagrandin E2	C20H32O5	7.49	351.00	[M–H]–	271.30	0.114	0.231
25	15-keto Prostaglandin E2	C20H30O5	7.55	349.00	[M–H]–	287.25	0.249	0.121
Docosahexanoic acid derived oxylipins								
26	(±)4-HDHA	C22H32O3	11.75	343.00	[M–H]–	101.05	0.018	0.042
27	(±)7-HDHA	C22H32O3	11.43	343.00	[M–H]–	141.15	0.004	0.012
28	(±)8-HDHA	C22H32O3	11.51	343.00	[M–H]–	189.25	0.028	0.074
29	(±)10-HDHA	C22H32O3	11.27	343.00	[M–H]–	153.00	0.045	0.075
30	(±)13-HDHA	C22H32O3	11.17	343.00	[M–H]–	193.20	0.019	0.051
31	14-HDHA	C22H32O3	11.75	343.00	[M–H]–	281.00	0.062	0.111
32	(±)16-HDHA	C22H32O3	11.09	343.00	[M–H]–	233.25	0.038	0.092
33	(±)17-HDHA	C22H32O3	11.43	343.00	[M–H]–	201.25	0.023	0.049

Eicosapentaenoic acid derived oxylipins								
34	8-HEPE	C20H30O3	10.67	317.20	[M–H]–	255.00	0.572	0.306
35	11-HEPE	C20H30O3	10.52	317.20	[M–H]–	167.00	0.090	0.501
36	(±)12-HEPE	C20H30O3	10.68	317.20	[M–H]–	179.20	0.442	0.456
37	(±)15-HEPE	C20H30O3	10.51	317.20	[M–H]–	219.20	0.087	0.375
38	(±)18-HEPE	C20H30O3	10.26	317.20	[M–H]–	255.25	0.108	0.678
39	(±)11(12)-EpETE	C20H30O3	12.22	317.20	[M–H]–	255.30	0.080	0.165
40	Prostaglandin D3	C20H30O5	7.56	349.20	[M–H]–	189.25	0.575	0.352
41	Prostaglandin E3	C20H30O5	5.70	349.20	[M–H]–	269.25	0.683	0.458
Dococosapentaenoic acid derived oxylipins								
42	17-HpDPA	C22H34O4	8.50	361.20	[M–H]–	221.10	0.293	0.898
Linoleic acid derived oxylipins								
43	9-HODE	C18H32O3	11.98	341.00	[M+HCOO]–	301.00	0.212	0.472
44	9-oxoODE	C18H30O3	11.67	293.00	[M–H]–	185.00	0.194	0.035
45	9(10)-EpOME	C18H32O3	10.95	295.00	[M–H]–	277.40	0.253	0.167
46	9,10-DiHOME	C18H34O4	9.46	313.00	[M–H]–	201.20	0.030	0.410
47	13-HODE	C18H32O3	11.88	341.00	[M+HCOO]–	301.05	0.167	0.257
48	12,13-DiHOME	C18H34O4	9.30	313.00	[M–H]–	183.25	0.111	0.403
Alpha-linolenic acid derived oxylipins								
49	9-HOTrE	C18H30O3	10.16	293.00	[M–H]–	275.00	0.198	0.477
50	9-HpOTrE	C18H30O4	10.28	309.00	[M–H]–	291.00	0.153	0.016
51	13-HOTrE	C18H30O3	11.76	339.00	[M+HCOO]–	298.00	0.066	0.958

Table S3. Gene-specific primers for semi-quantitative RT-PCR analysis

Gene		Primer Sequence (5' to 3')
Il-6	Forward	AGT TGC CTT CTT GGG ACT GA
	Reverse	TCC ACG ATT TCC CAG AGA AC
Il1β	Forward	CTT CAG GCA GGC AGT ATC ACT C
	Reverse	TTG TTG TTC ATC TCG GAG CC
Tnf-α	Forward	AGC CCC CAG TGT GTA TCC TT
	Reverse	ACA GTC CAG GTC ACT GTC CC
Fizz1	Forward	CAA GGA ACT TCT TGC CAA TCC AG
	Reverse	CCA AGA TCC ACA GGC AAA GCC A
Mmp13	Forward	GAT GAC CTG TCT GAG GAA GAC C
	Reverse	GCA TTT CTC GGA GCC TGT CAA C
Muc5ac	Forward	CCA CTT TCT CCT TCT CCA CAC C
	Reverse	GGT TGT CGA TGC AGC CTT GCT T
Ym1	Forward	TCA CAG GTC TGG CAA TTC TTC TG
	Reverse	TTT GTC CTT AGG AGG GCT TCC TCG
Vegf	Forward	CTG CTG TAA CGA TGA AGC CCT G
	Reverse	GCT GTA GGA AGC TCA TCT CTC C
Cd206	Forward	GTT CAC CTG GAG TGA TGG TTC TC
	Reverse	AGG ACA TGC CAG GGT CAC CTT T
Cxcr4	Forward	GAC TGG CAT AGT CGG CAA TGG A
	Reverse	CAA AGA GGA GGT CAG CCA CTG A
Ccl24	Forward	ATT CCA GAA AAC CGA GTG GTT AGC
	Reverse	GCA TCC AGT TTT TGT ATG TGC CTC
Cxcl13	Forward	CAT AGA TCG GAT TCA AGT TAC GCC
	Reverse	GTA ACC ATT TGG CAC GAG GAT TC
Cyp4f13	Forward	CCC TAA ACC GAG CTG GTT CTG
	Reverse	GAG TCG CAG GAT TGG GTA CAC
Cyp4f39	Forward	CGA GCA CAT CAG CCT TAT GAC C
	Reverse	TCC AGG TAG TGA TGC AAG CGG T
Alox12	Forward	CTC TTG TCA TGC TGA GGA TGG AC
	Reverse	AAG AGC CAG GCA AGT GGA GGA
